# Supplementary material for: A common MET polymorphism harnesses HER2 signaling to drive aggressive squamous cell carcinoma
Source: Nat Commun. 2020 Mar 25;11:1556. doi: 10.1038/s41467-020-15318-5 (PMC7096530; doi:10.1038/s41467-020-15318-5)
Supplement: Supplementary file 1 — Supplementary Information [file 41467_2020_15318_MOESM1_ESM.pdf]

A common *MET* polymorphism harnesses HER2 signaling to drive aggressive squamous cell carcinoma

Kong et al.

SUPPLEMENTARY INFORMATION

SUPPLEMENTARY METHODS

Clinical specimens and data

Human formalin-fixed paraffin-embedded (FFPE) tumor specimens and tissue microarrays were obtained from Department of Pathology, National University Hospital (NUH, Singapore), as well as from patients who have undergone surgical resection at NUH. Germline DNA was obtained retrospectively from Pharmacogenetics DNA Bank and prospectively from patients with metastatic LUSC, HNSCC and NPC. All samples were processed with approval from National Healthcare Group's Domain Specific Review Board (DSRB) Committee, and under the guideline of Institutional Review Board (IRB) at National University of Singapore (NUS, Singapore). Written informed consent was obtained in all cases from patients at time of enrolment.

Mass spectrometry analysis.

Samples were treated by in-gel digestion prior to MS analysis. In brief, samples were reduced in 10 mM DTT for 1h at 56°C followed by alkylation with 55mM iodoacetamide (Sigma) for 45 min in the dark. Tryptic digest was performed in 50 mM ammonium bicarbonate buffer with 2 µg trypsin at 37°C overnight. Peptides were desalted on StageTips and analysed by nanoflow liquid chromatography on an EASY-nLC 1200 system coupled to a Q Exactive HF mass spectrometer (Thermo Fisher Scientific). Peptides were separated on a C18-reversed phase column (25 cm long, 75 µm inner diameter) packed in-house with ReproSil-Pur C18-QAQ 1.9 µm resin (Dr Maisch). The column was mounted on an Easy Flex Nano Source and temperature controlled by a column oven (Sonation) at 40°C. A 105-min gradient from 2 to 40% acetonitrile in 0.5% formic acid at a flow of 225 nl/min was used. Spray voltage was set to 2.4 kV. The Q Exactive HF was operated with a TOP20 MS/MS spectra acquisition method per MS full scan. MS scans were conducted with 60,000 and MS/MS scans with 15,000 resolution. The raw files were processed with MaxQuant version 1.5.2.8 [1] with preset standard settings for SILAC labelled samples and the re-quantify option was activated. Carbamidomethylation was set as fixed modification while methionine oxidation and protein N-acetylation were considered as variable modifications. Search results were filtered with a false discovery rate of 0.01.

Cell viability assay.

Approximately 3,000 cells/well in complete medium were seeded into clear-bottom black 96-well plates 24 hours prior to drug treatment. Indicated compounds or rSema were added in serial dilution for 72 hours. The viability of cells were assayed using CellTiter-Glo luminescent reagent (Promega, #G7572). The luminescence signals were detected using TECAN Infinite M1000 pro multi-mode plate reader using an integration time of 1000 ms. The relative luminescence units from treated wells were normalized against the readings from DMSO control and expressed as percentage cell viability and presented as mean ± SD relative to DMSO-treated controls. IC_{50} values were calculated by curve-fitting using GraphPad Prism software, and expressed as mean value ± SD.

RNAseq, EMT scoring, hierarchical clustering and pathway analysis.

RNAseq and gene expression analyses were performed by Novogene. In brief, isogenic H2170 cell lines of wild type (WT) and MET^{N375S} genotypes were subjected to Illumina HiSeqTM2500 for RNA-seq. CASAVA v1.8 was used to convert the raw data to fastq files. Reads of low quality, adapter contamination, and reads with undetectable bases more than 10% were removed, yielding 38,860,951 and 35,817,538 reads for WT and N375S respectively. Subsequently, we applied TopHat2 to map the reads to Ensembl human genome GRCh37. Only uniquely mapped reads for both WT 67,802,021 (87.24%) and N375S 62,325,393 (87%) were retained for further analyses. The read counts were then converted to FPKM by normalizing the read counts by transcript length. Differentially expressed genes were identified using R package DESeq. Genes with adjusted p-value < 0.05 were deemed significant and selected for further analyses. Differentially expressed genes were subjected to GO enrichment analyses using GOseq and KEGG pathway enrichment analyses. Epithelial-mesenchymal transition (EMT) enrichment score was computed using a signature and a two-sample Kolmogorov-Smirnov-based method [2], with a negative score (<0) indicates epithelial-like and positive score (>0) indicates mesenchymal-like phenotypes. GEO Accession number: GSE128956.

Droplet digital Polymerase Chain Reaction (ddPCR)

Genomic DNA (gDNA) was extracted from patient's whole blood and plasma using QIAamp DNA mini kit (Qiagen, #51306) and QIAamp DNA FFPE Tissue kit (Qiagen, #56404) according to the manufacturer's protocols. 20 ng of gDNA (or circulating DNA from 100 µl of plasma) was used to perform ddPCR on Bio-Rad QX200TM Droplet Digital PCR system for detection of single nucleotide polymorphism of *MET* gene. Data was presented as hetero- or homozygous for *MET (N375S)* variant. Sequences of primers and probes are shown in Supplementary Table 3.

Targeted exome sequencing

Amplicon-enriched targeted sequencing was performed and reported previously [3]. Library preparation was conducted using GeneRead DNAseq Gene Human Lung Panel (Qiagen) according to manufacturer's protocol, and exome sequencing was performed on Illumina HiSeq2000 Platform. The generated sequencing reads were subjected to variant calling by Genome Analysis Toolkit (GATK) from Broad Institute. Significantly mutated genes ($P < 0.01$) were identified by mapping to public database (dbSNP) and subjected to functional annotation with the COSMIC Database for detection of cancer-related mutations. Alterations on the *MET, EGFR, KRAS, BRAF* and *ERBB2* genes were reported in this study. BioProject ID: PRJNA529714.

Molecular Dynamics Simulations of MET Sema domain.

The crystal structure of the Sema domain of wildtype MET (PDB ID: 4K3J) was used to explore the effects of the N375S mutation on the conformational effects. The missing loops/residues were modelled using Modeller [4]. The structure of MET^{N375S} was generated by mutating Asn375 to Ser in the MET^{wt} structure using Pymol Program. These structures (of both MET^{wt} and MET^{N375S}) were subjected to molecular dynamics (MD) simulations using standard protocols and the program Amber18 [5]. To model possible modes of interactions between c-MET and HER2, we utilized the program CLICK to compare structural regions between two different proteins, irrespective of sequence similarity. This search was performed on the conformations of MET^{wt} and MET^{N375S} that were generated during the MD simulations. This search yielded a good conformational match between the region

encompassing residues 364 to 385 in the Sema domain (the region colored as orange/magenta in the Supplementary Figure 12) with the structure of EGF in its complex with EGFR (PDB ID: 3LTF). The homology between the structures of EGFR and HER2 was used to guide the design of a complex between MET^{wt} and HER2 and between MET^{N375S} and HER2 and these complexes were subject to MD simulations.

REFERENCES

1 Cox, J. & Mann, M. MaxQuant enables high peptide identification rates, individualized p.p.b.-range mass accuracies and proteome-wide protein quantification. *Nat Biotechnol* **26**, 1367-1372, doi:10.1038/nbt.1511 (2008).

2 Tan, T. Z. *et al.* Epithelial-mesenchymal transition spectrum quantification and its efficacy in deciphering survival and drug responses of cancer patients. *EMBO Mol Med* **6**, 1279-1293, doi:10.15252/emmm.201404208 (2014).

3 Kong, L. R. *et al.* MEK Inhibition Overcomes Cisplatin Resistance Conferred by SOS/MAPK Pathway Activation in Squamous Cell Carcinoma. *Mol Cancer Ther* **14**, 1750-1760, doi:10.1158/1535-7163.MCT-15-0062 (2015).

4 Sali, A. & Blundell, T. L. Comparative protein modelling by satisfaction of spatial restraints. *J Mol Biol* **234**, 779-815, doi:10.1006/jmbi.1993.1626 (1993).

5 Kannan, S. *et al.* Hydration effects on the efficacy of the Epidermal growth factor receptor kinase inhibitor afatinib. *Sci Rep* **7**, 1540, doi:10.1038/s41598-017-01491-z (2017).

Supplementary Figure 1

A

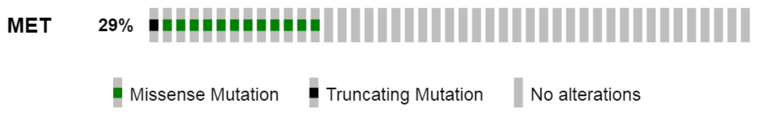

B

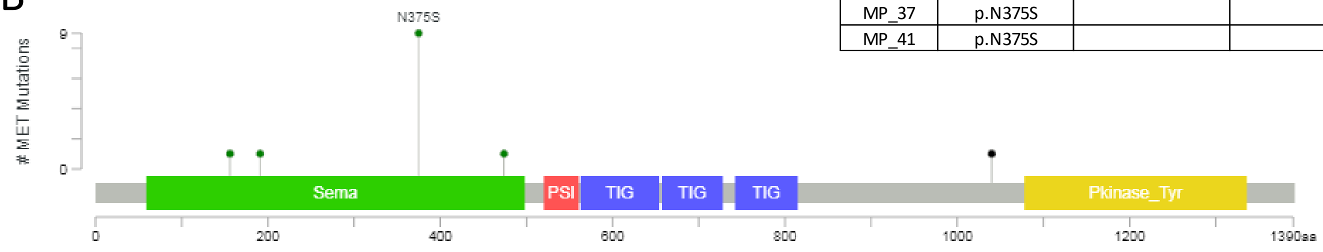

C

Sample ID	*MET* alteration	*EGFR* alteration	*BRAF* alteration
MP_04	p.N375S		
MP_06	p.N375S		
MP_11	p.N375S		
MP_16	p.N375S	p.C311Y, p.G779D, p.L861Q, p.S811F	p.I582M
MP_24	p.N375S	p.G721D	
MP_26	p.N375S		
MP_32	p.N375S		
MP_37	p.N375S		
MP_41	p.N375S		

Supplementary Fig. 1: Mutations on *MET* gene in LUSC tissue specimens.
(A-B) Mutations on *MET* gene as sequenced by amplicon-enriched NGS. Oncoprint (A) and lollipop plot (B) demonstrating the distribution of missense/nonsense mutations across the 45 FFPE LUSC specimens.
(C) Tabulation of other genetic alterations (*EGFR*, *BRAF*) found in the MET^{N375S}-positive tumors. No alteration was detected on the *KRAS* and *ERBB2* genes among these samples.

Supplementary Figure 2

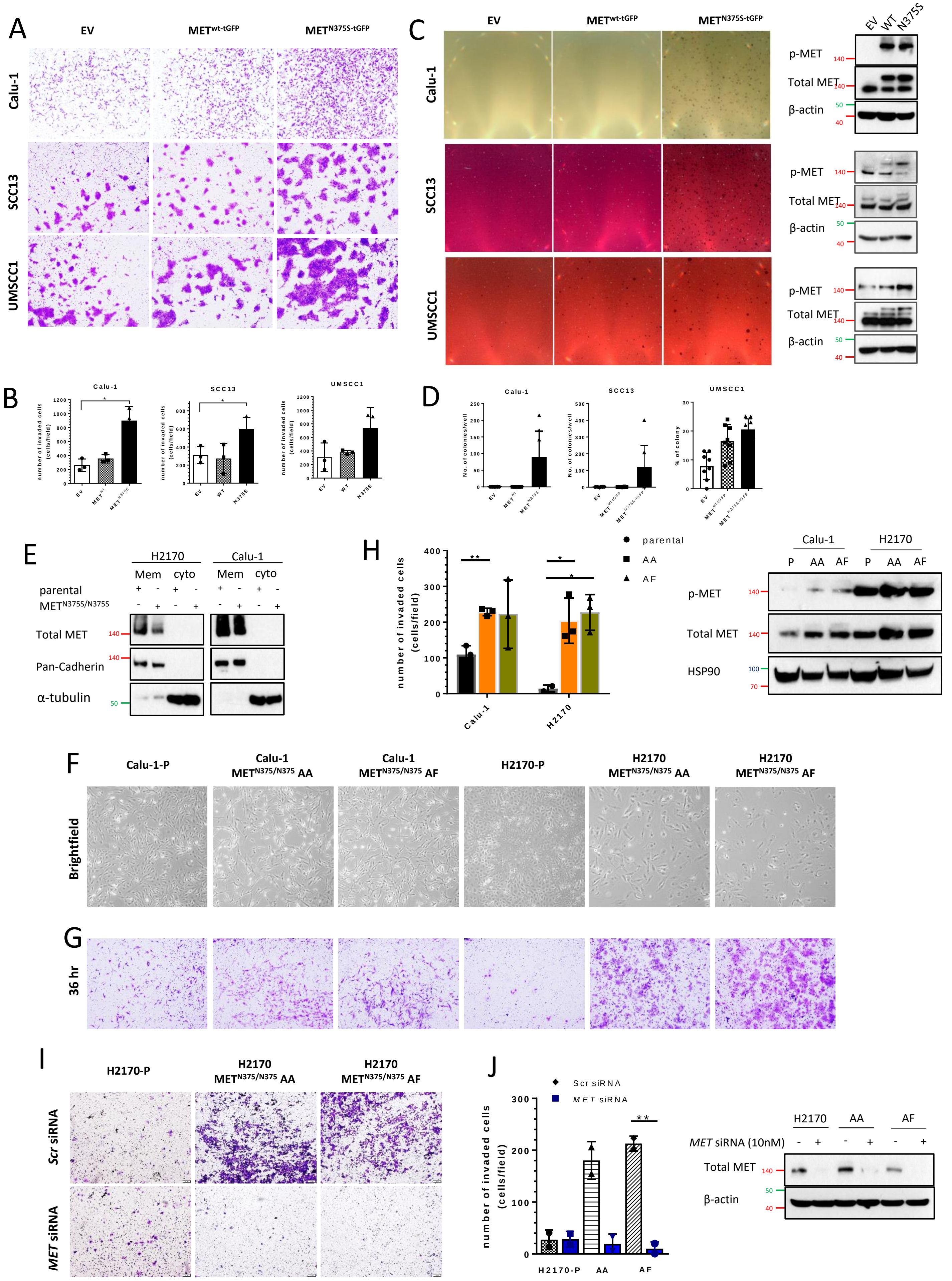

Supplementary Fig. 2: Polymorphic N375S variant enhances aggressive tumor phenotype in LUSC and HNSCC cells.

(A-D) Cell invasion and colony forming assays were performed on isogenic LUSC (Calu-1) and HNSCC (SCC13, UMSCC1) cells transfected with empty vector (EV), wild-type ($MET^{wt\text{-}tGFP}$) and N375S mutant ($MET^{N375S\text{-}tGFP}$). Immunoblots demonstrated expression levels of total and phosphorylated MET were shown (far right). β-actin was used as loading control.

(A-B) For invasion assay, cells seeded in Matrigel invasion chambers were fixed and stained at 36 hours. (A) Representative images were shown. (B) The number of cells in four random microscopic fields (40×) were quantified for EV, $MET^{wt\text{-}tGFP}$ and $MET^{N375S\text{-}tGFP}$ cells and expressed as mean ± SD (n = 2-3). Two tailed Student's t-test, $*P < 0.05$.

(C-D) Representative images of colony formation for each cell types, stained with MTT at assay endpoint (C). (D) The number of colonies were quantified for EV, $MET^{wt\text{-}tGFP}$ and $MET^{N375S\text{-}tGFP}$ cells and expressed as mean of triplicates ± SD ($n > 3$).

(E) Immunoblot analysis of membrane and cytosolic fractions were performed for the CRISPR-edited, $MET^{N375S/N375S}$ homozygous Calu-1 and H2170 cells cell lines. Pan-Cadherin (membrane) and α-tubulin (cytoplasmic) were used as loading controls.

(F-J) Cellular properties of $MET^{N375S/N375S}$ cells were shown. Immunoblots demonstrated expression levels of total and phosphorylated MET were shown (right side of Figure S2H). β-actin was used as loading control. (F) Representative images of the morphology of CRISPR-edited Calu-1 and H2170 clones (AA and AF). (G) Invasion assays were performed on the $MET^{N375S/N375S}$ H2170 and Calu-1 clones. Representative images were shown. (H) The number of invaded cells in four random microscopic fields (40×) were quantified and expressed as mean ± SD (n = 3). (I) Invasion assays were performed on the $MET^{N375S/N375S}$ H2170 clones transfected with *Scr* or *MET* siRNA. Immunoblots demonstrated expression levels of total and phosphorylated MET were shown (bottom right). HSP90 was used as loading control. Representative images were shown. (J) The number of invaded cells in four random microscopic fields (40×) were quantified and expressed as mean ± SD (n = 2). Two tailed Student's t-test, $*P < 0.05$; $**P < 0.01$.

Supplementary Figure 3

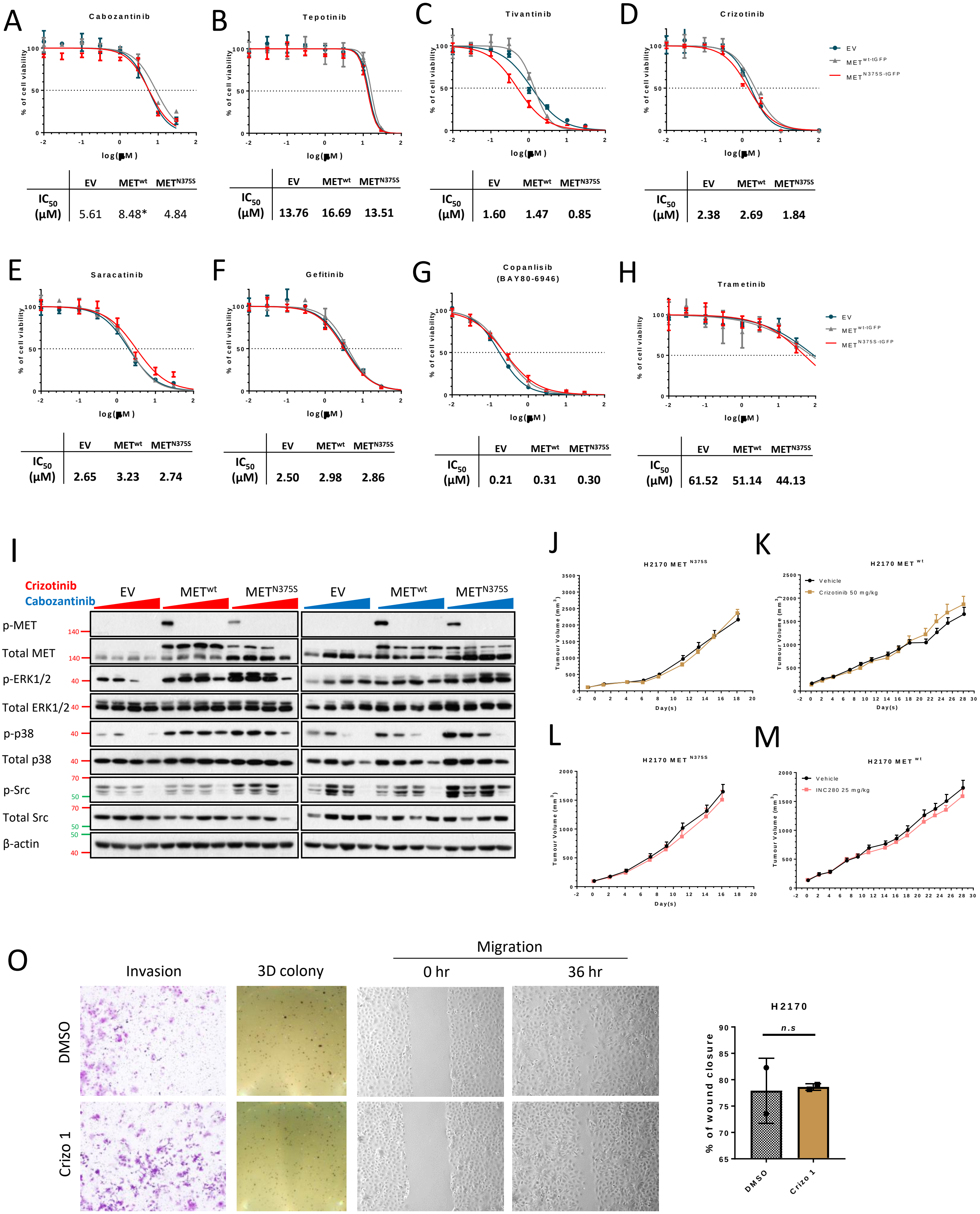

Supplementary Fig. 3: Forced expression of MET^{N375S} does not impact susceptibility to MET inhibitors.

(A-H) Cell viability assays comparing sensitivities of isogenic H2170 cells to various TKIs. Cells were treated with increasing concentrations (0.01 to 30 μM) of cabozantinib (A), tepotinib (B), tivantinib (C), crizotinib (D), saracatinib (E), gefitinib (F), copanlisib (G), and trametinib (H) for 72 hours, and assayed with CellTiter-Glo reagents. Each dose-response curve showing the mean of triplicates ± SD for a representative experiment. Below, mean IC_{50} for the respective cell lines ($n = 3$). Two tailed Student's t-test; $*P < 0.05$.

(I) H2170 cells with ectopic expression of MET were treated with crizotinib or cabozantinib (1, 3, 10 μM). Immunoblots showing expression of the indicated targets in lysates of the isogenic H2170 EV, $MET^{wt\text{-}tGFP}$ and $MET^{N375S\text{-}tGFP}$ cells. Cells were harvested 48 hours after treatment, and β-actin was used as loading control.

(J-M) Efficacies of MET inhibitors were evaluated in xenograft models. Tumor growth of MET^{wt} (J) and MET^{N375S} (K) xenografts after treatment with crizotinib; and of MET^{wt} (L) and MET^{N375S} (M) xenografts after treatment with INC280 were expressed at mean ± SEM ($n = 5$).

(O) H2170 $MET^{N375S\text{-}tGFP}$ cells were treated with 1 μM crizotinib or 0.1% DMSO for 24hours, and subjected to cell invasion ($n = 3$), colony forming ($n = 3$), and cell migration ($n = 2$) assays. The wound closure percentages were expressed as mean ± SD. Two tailed Student's t-test; β-actin was used as loading control.

Supplementary Figure 4

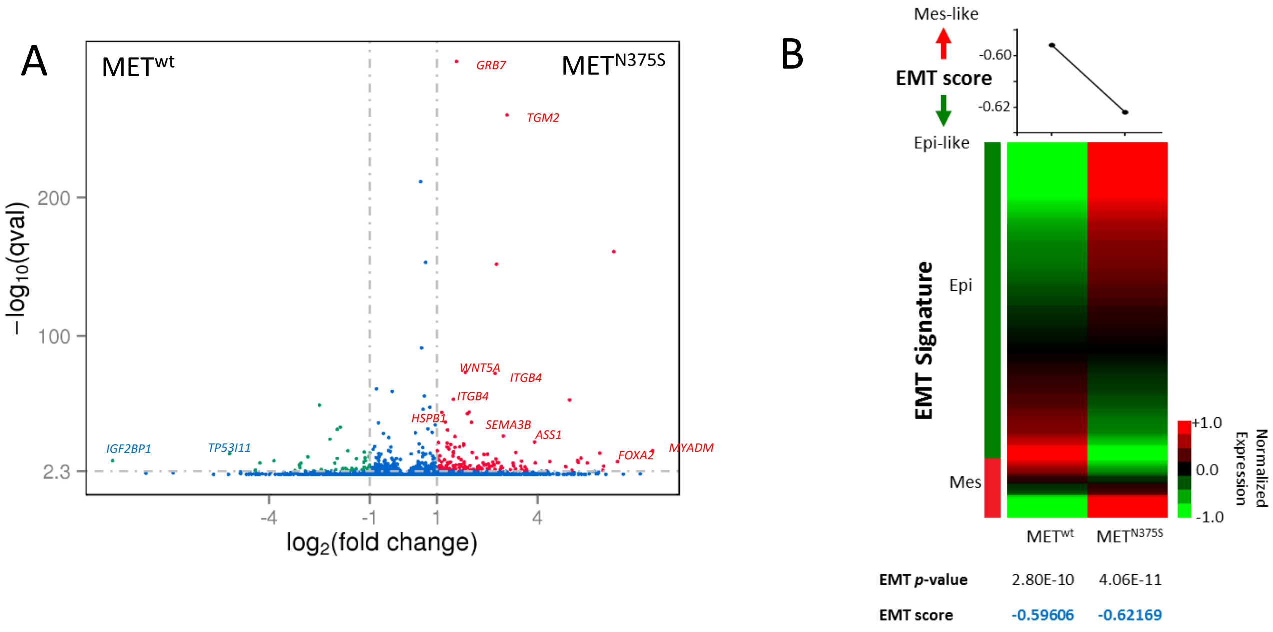

Supplementary Fig. 4: Comparative transcriptomic of H2170 MET^{wt} and MET^{N375S} cells.
(A) RNAseq was performed on total RNA harvested from isogenic H2170 MET$^{wt\text{-}tGFP}$ and MET$^{N375S\text{-}tGFP}$ cells. Volcano plot presentation of differentially expressed genes in the WT versus N375S cells. Red and blue points marked the up-regulated and down-regulated genes respectively in MET$^{N375S\text{-}tGFP}$ cells. The x-axis, $\log_{2(}$fold-changes) in expression; y-axis, -$\log_{10}$(q-value).
(B) Heatmap comparing transcriptomic changes in epithelial-mesenchymal transition (EMT) gene signature between MET$^{wt\text{-}tGFP}$ and MET$^{N375S\text{-}tGFP}$ cells. EMT scores documenting the interplays between epithelial and mesenchymal genes. Epi, epithelial; Mes, mesenchymal.

Supplementary Figure 5

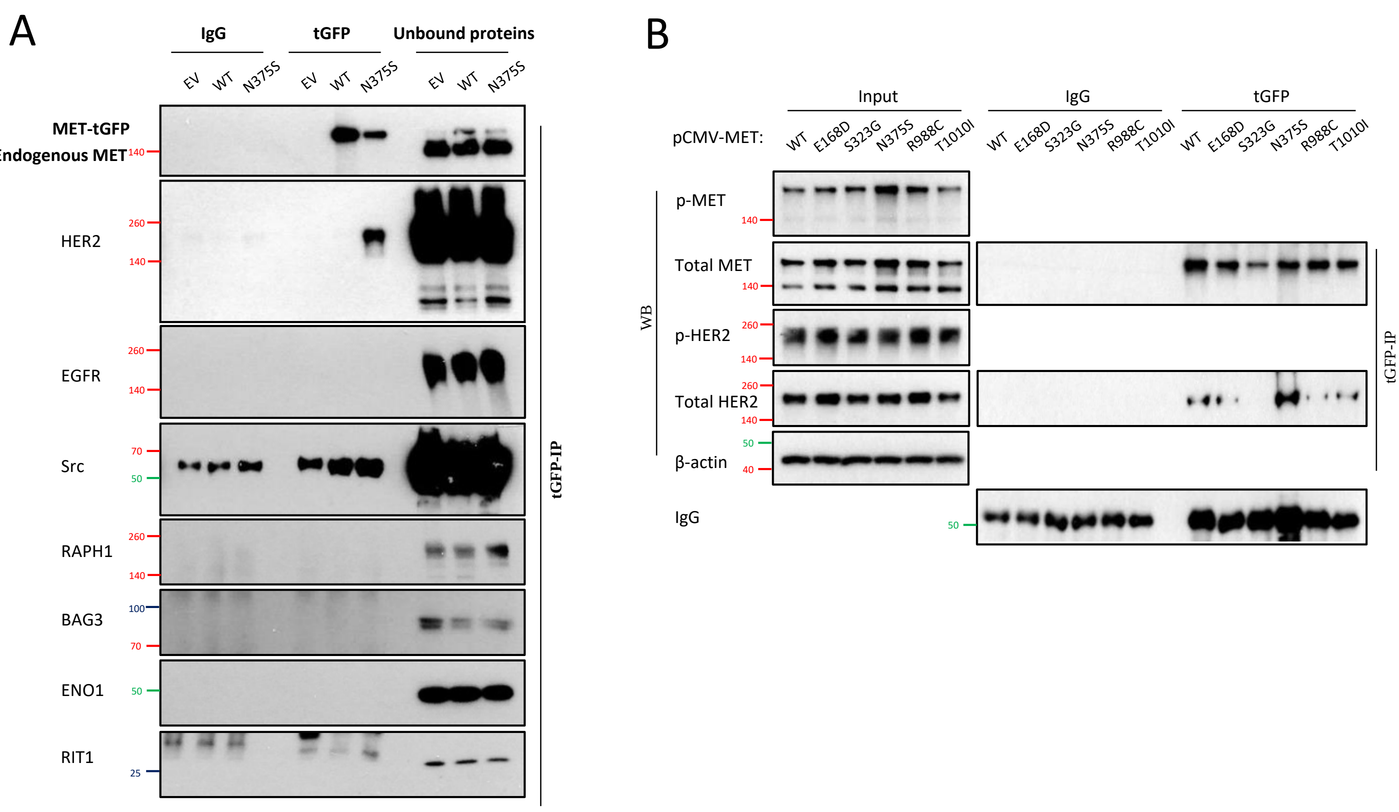

Supplementary Fig. 5: Validating the specificity of MET^{N375S}-HER2 interaction.

(A) Interaction of ectopic MET and endogenous HER2 in H2170 $MET^{wt\text{-}tGFP}$ and $MET^{N375S\text{-}tGFP}$ cells was detected with immunoprecipitation and immunoblotting. Targets identified from SILAC experiment were validated (BAG3, RAPH1, ENO1 and RIT1) together with EGFR and Src. Representative blots of three independent experiments was shown (n = 3).

(B) Specificity of MET^{N375S} interaction with HER2 was compared to other common MET variants. HEK293 cells were transfected with 1 µg of either pCMV6-EV vector, MET-wt, E168D, S323G, N375S, R988C, or T1010I, together with pCMV6-ERBB2-DDK plasmid, for 24 hours. MET/HER2 interaction in HEK293 cells was detected with immunoprecipitation and immunoblotting. Left, input controls and phosphorylated proteins. β-actin was used as loading control.

Supplementary Figure 6

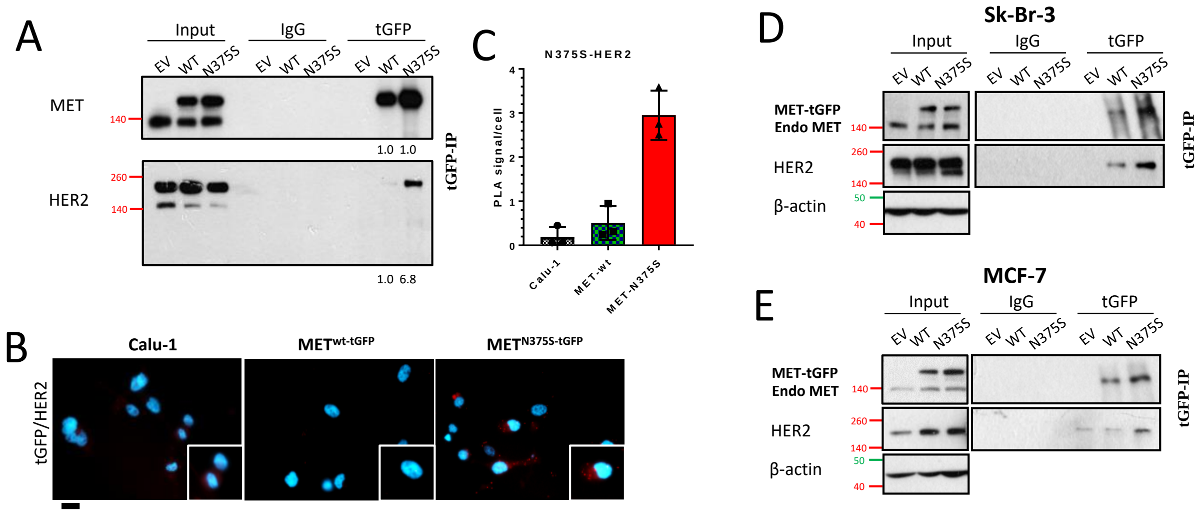

Supplementary Fig. 6: Association of MET^{N375S} and HER2 in lung and breast carcinoma cells.
(A) Interaction of ectopic MET and endogenous HER2 in Calu-1 MET$^{wt\text{-}tGFP}$ and MET$^{N375S\text{-}tGFP}$ cells was detected with immunoprecipitation and immunoblotting. Left, input controls. Total MET and HER2 band intensities, normalized to input controls and relative to MET^{wt}, are shown below. Values represent average of three independent experiments.
(B-C) Detection of MET/HER2 co-localization (red) in EV, MET$^{wt\text{-}tGFP}$, and MET$^{N375S\text{-}tGFP}$ cells with proximity ligation assay (PLA). Representative images were shown (B), with the PLA signals quantified (C) and expressed as number of signals/cell ± SD (n = 3). Scale bar, 20 µm.
(D-E) MET/HER2 interaction was validated in breast carcinoma cell lines, Sk-Br-3 (HER2^{+}) (D) and MCF-7 (HER2^{-}) (E), by immunoprecipitation and immunoblotting after transient transfection of pCMV6-MET (wt) and pCMV6-MET (N375S). 1 µg of plasmid was used per transfection. β-actin was used as loading control.

Supplementary Figure 7

A

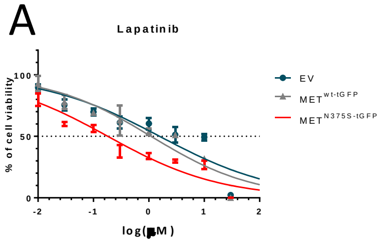

	EV	MET^{wt}	MET^{N375S}
IC_{50} (µM)	2.17	1.34	0.27*

B

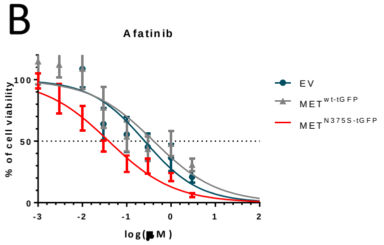

	EV	MET^{wt}	MET^{N375S}
IC_{50} (µM)	0.20	0.30	0.07*

C

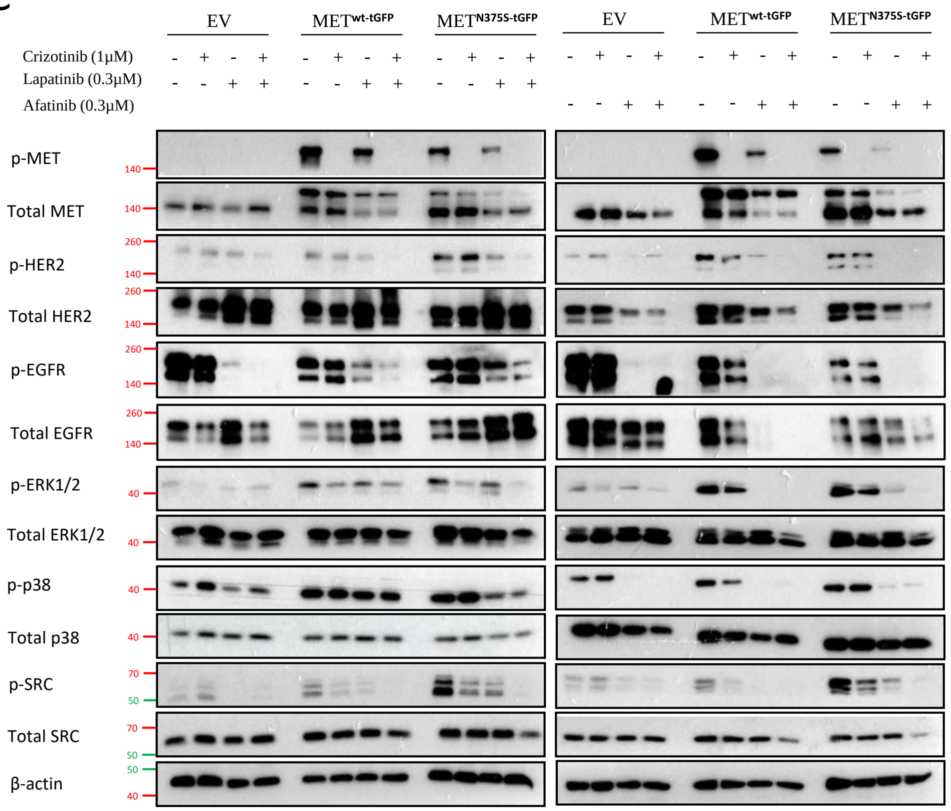

Supplementary Fig. 7: Forced expression of MET^{N375S} increases susceptibility to HER2 inhibitors. (A-B) Cell viability assays showing sensitivities of isogenic H2170 cells to HER2 inhibitors. Cells were treated with increasing concentrations of lapatinib (A) and afatinib (B) for 72 hours, and assayed with CellTiter-Glo reagents. Each dose-response curve showing the mean of triplicates ± SD for a representative experiment. Below, mean IC_{50} for the respective cell lines ($n = 3$). Two tailed Student's t-test; $*P < 0.05$. (C) H2170 cells with ectopic expression of MET were treated with crizotinib (1 µM), lapatinib (0.3 µM), afatinib (0.3 µM), or the indicated combinations. Immunoblots showing expression of the indicated targets in lysates of the isogenic H2170 EV, $MET^{wt\text{-}tGFP}$ and $MET^{N375S\text{-}tGFP}$ cells. Blots are representative of three independent experiments. Cells were harvested 48 hours after treatment, and β-actin was used as loading control.

Supplementary Figure 8

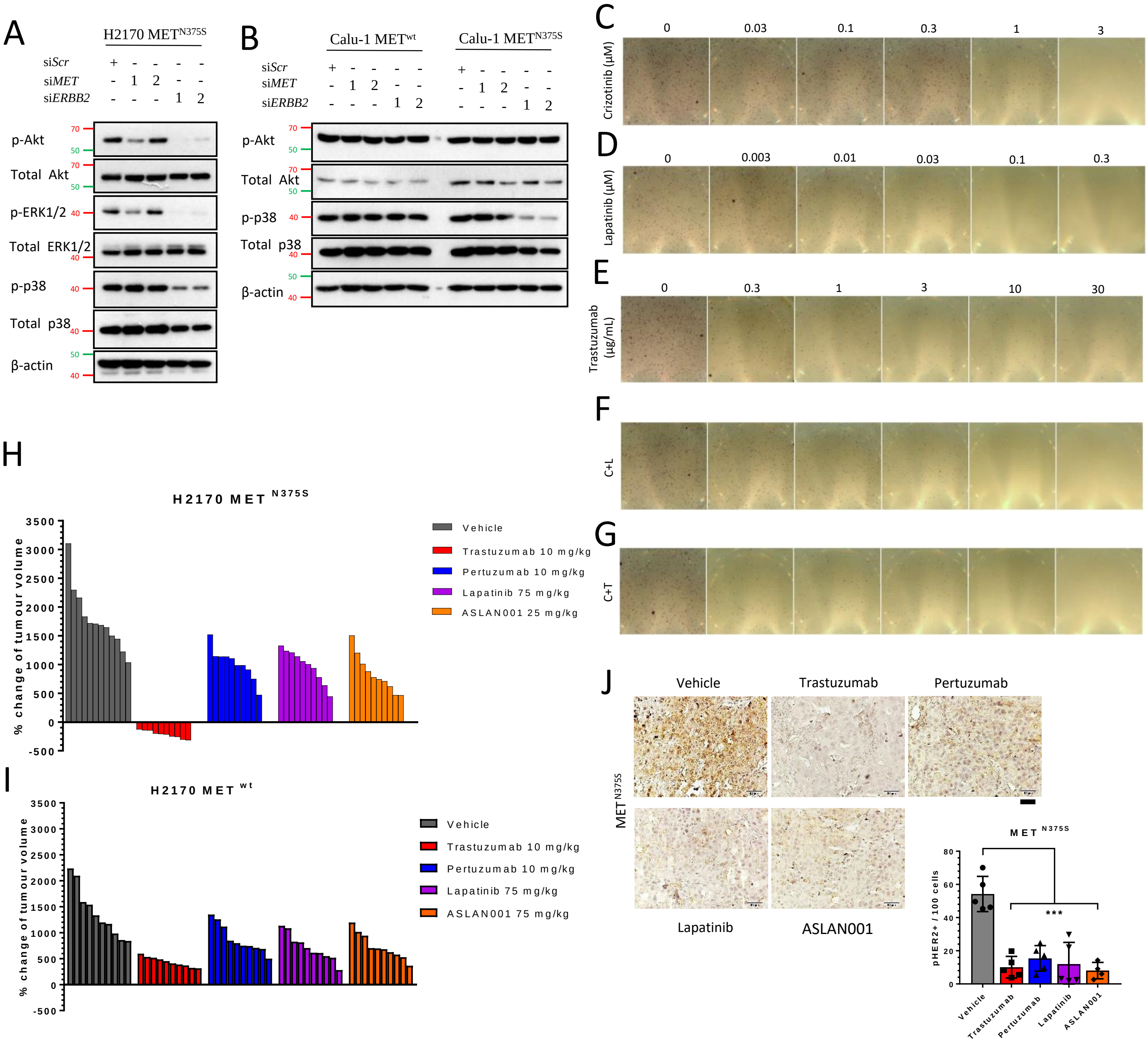

Supplementary Fig. 8: HER2 inhibition demonstrates anti-SCC efficacy in MET^{N375S} cells.
(A-B) H2170 and Calu-1 cells were co-incubated with 10 µM of the indicated siRNA for 48 hours. Immunoblots showing changes on the indicated targets by *MET* and *ERBB2* silencing in H2170 $MET^{N375S\text{-}tGFP}$ (A), Calu-1 $MET^{wt\text{-}tGFP}$ and $MET^{N375S\text{-}tGFP}$ cells (B). Blots are representative of two independent experiments. β-actin was used as loading control.
(C-G) Anchorage-independent colony formation on isogenic H2170 $MET^{N375S\text{-}tGFP}$ cells, treated with the indicated doses of crizotinib (C), lapatinib (D), trastuzumab (E), crizotinib/lapatinib (C/L) combination (F), or crizotinib/trastuzumab (C/T) combination (G) for 4 weeks. Representative images were shown for three independent experiments.
(H-J) Efficacies of HER2 inhibitors were evaluated in xenograft models. Waterfall plots comparing tumor growth of MET^{wt} (H) and MET^{N375S} (I) xenografts after treatment with trastuzumab, pertuzumab, lapatinib and ASLAN001. (J) Immunohistochemistry staining showing the changes in p-HER2 after treatment with the respective inhibitors in and MET^{N375S} tumors. Representative images were shown. Scale bar, 50 µm. Right, expression of p-HER2 was quantified and expressed at mean of positive-staining/100 cells ± SD (*n* = 5). Two tailed Student's t-test, **P* < 0.05, ***P* < 0.01, ****P* < 0.001.

Supplementary Figure 9

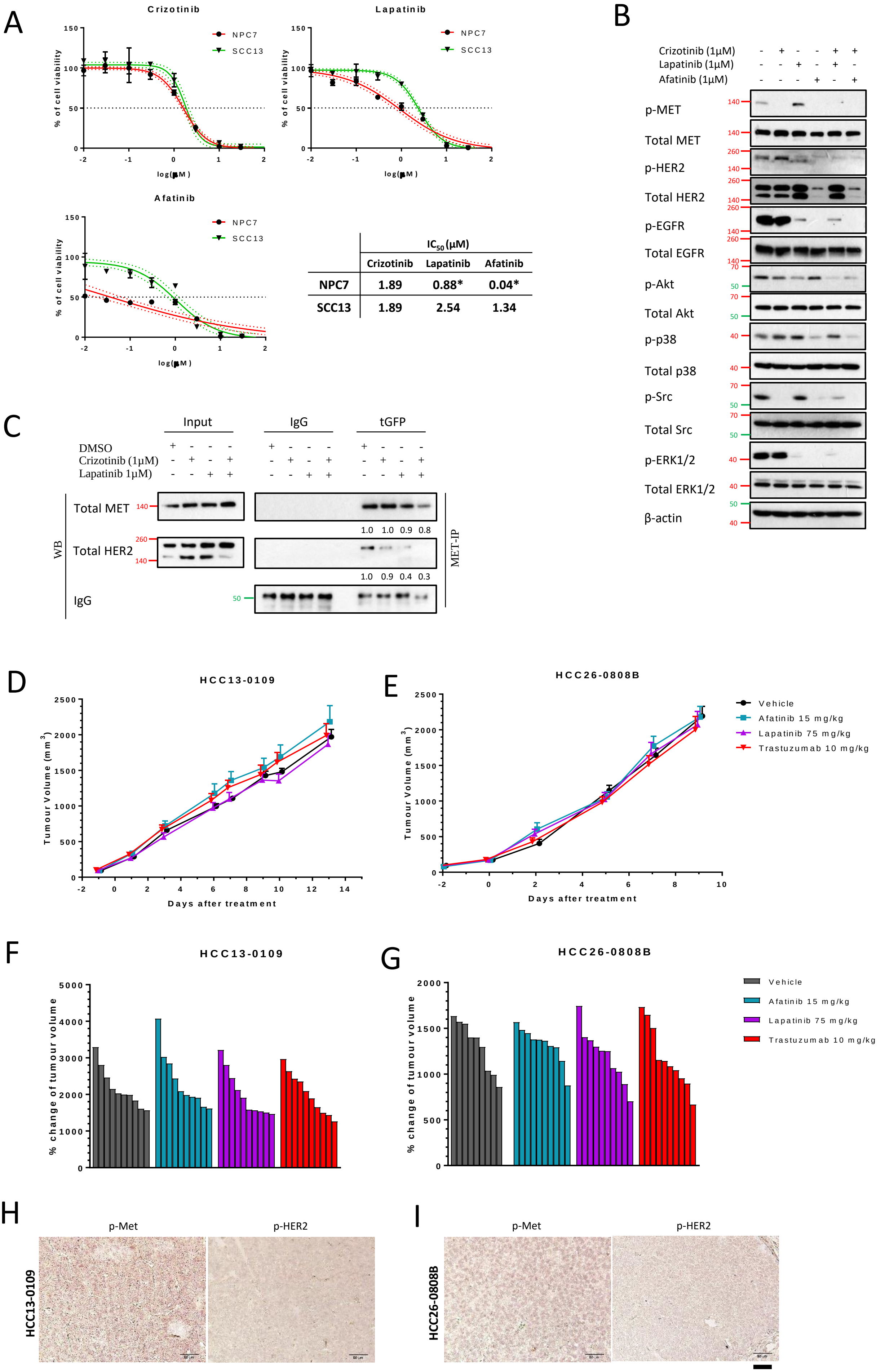

	IC50 (μM)		
	Crizotinib	Lapatinib	Afatinib
NPC7	1.89	0.88*	0.04*
SCC13	1.89	2.54	1.34

Supplementary Fig. 9: HER2 inhibitors demonstrate anti-SCC properties in HNSCC cells but show lack of efficacy in HCC tumors.
(A) Cell viability assays showing sensitivities of NPC7 primary culture and SCC13 cell line to crizotinib, lapatinib and afatinib. Cells were treated with increasing concentrations of the compound for 72 hours, and assayed with CellTiter-Glo reagents. Each dose-response curve showing the mean of triplicates ± SD for a representative experiment. Below, mean IC_{50} for the respective cell lines ($n = 3$). Two tailed Student's t-test; $*P < 0.05$.
(B-C) NCC-NPC7 (HNSCC primary cells) was treated with crizotinib (1 µM), lapatinib (1 µM), afatinib (1 µM), or in the indicated combinations for 48 hours. Immunoblots showing changes on the indicated targets (B), while interaction of MET and HER2 in NCC-NPC7 cells, after treatment with vehicle (0.1% DMSO), MET inhibitor crizotinib (1 µM), HER2 inhibitor lapatinib (1 µM), or crizotinib/lapatinib combination, was detected with immunoprecipitation and immunoblotting (C). Total MET and HER2 band intensities, relative to vehicle treated group, are shown below. Values represent average of two independent experiments. Left, input controls. β-actin or IgG was used as loading control.
(D-I) Efficacies of HER2 inhibitors were evaluated in patient-derived xenograft (PDX) models of hepatocellular carcinoma (HCC) expressing MET^{N375S} variant. Tumor growth of HCC13-0109 (D) and HCC26-0808B (E) xenografts after treatment with lapatinib, afatinib and trastuzumab were expressed at mean ± SEM ($n = 5$). Waterfall plots comparing tumor growth of HCC13-0109 (F) and HCC26-0808B (G). Immunohistochemistry staining showing the expression of p-MET and p-HER2 in HCC13-0109 (H) and HCC26-0808B (I). Representative images were shown. Scale bar, 50 µm.

Supplementary Figure 10

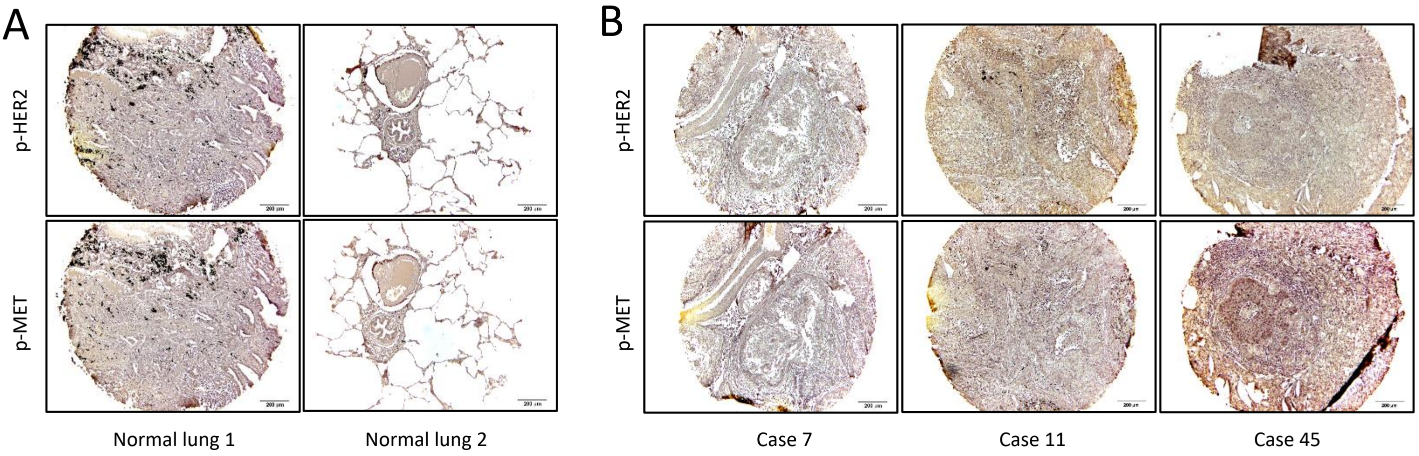

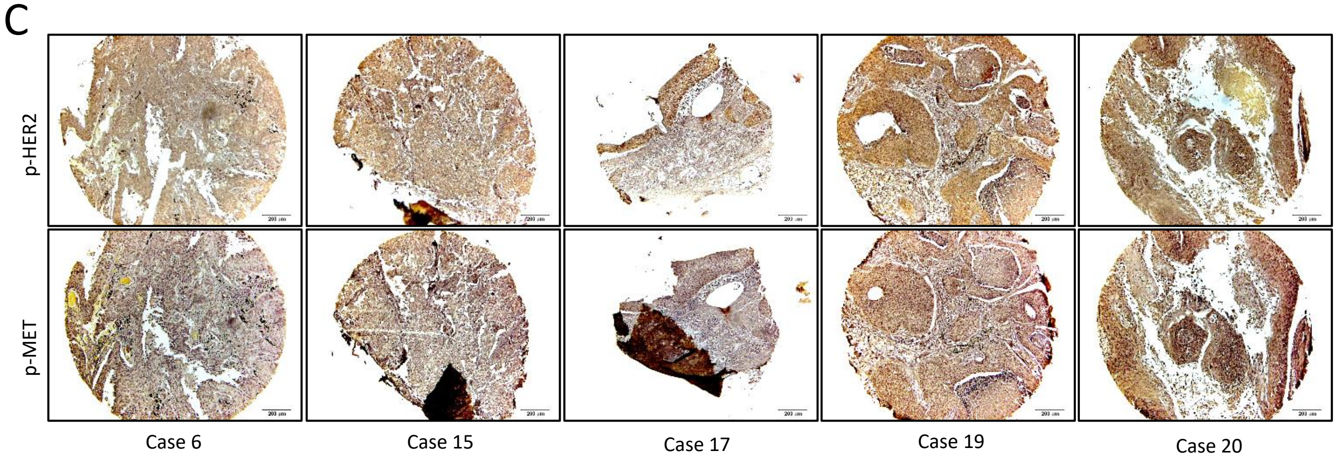

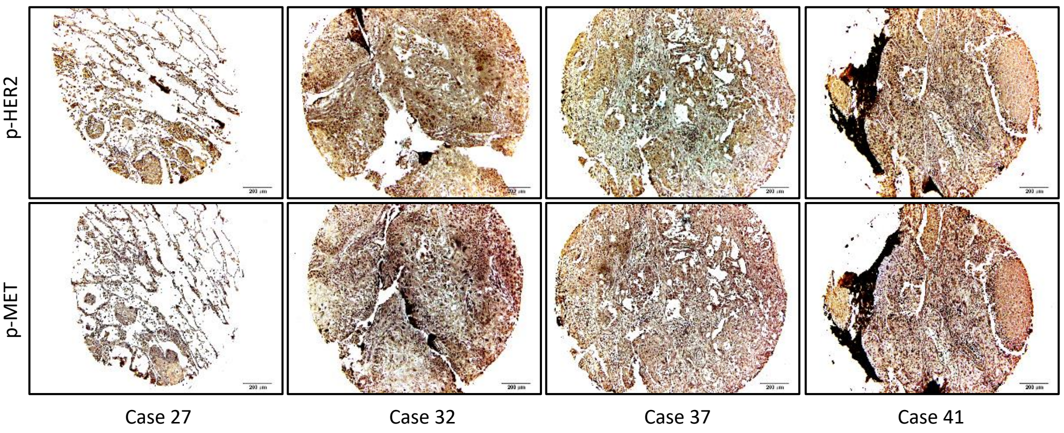

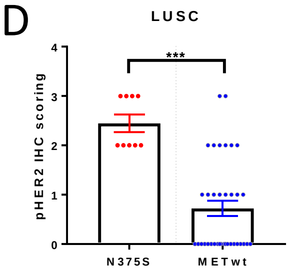

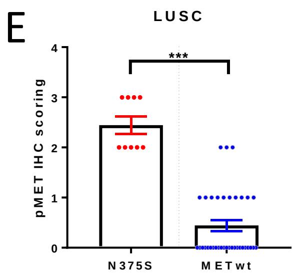

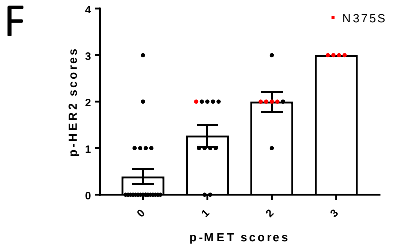

		p-MET score	
		Low (0-1)	High (2-3)
p-HER2 score	Low (0-1)	27	1
	High (2-3)	7	10

P < 0.0001

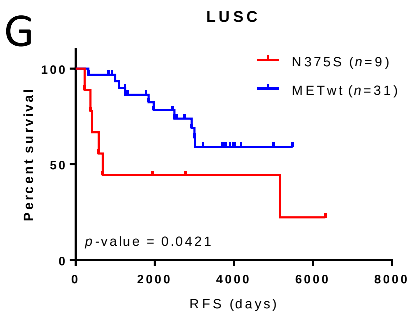

Supplementary Fig. 10: MET (N375S) status is positively correlating with p-HER2 expression in LUSC.

(A-F) Immunohistochemistry staining showing the expression of p-MET and p-HER2 in normal lung tissues (A), MET^{wt} LUSC tumors (*n* = 36) (B) and MET^{N375S}-positive LUSC tumors (*n* = 9) (C). Representative images were shown. Scale bar, 200 μm. Scoring of individual tumor was tabulated in Table S2, and plotted as bar charts for p-HER2 (D) and p-MET (E). The correlation between p-HER2 and p-MET associated with MET polymorphism was plotted (F). Data were expressed at mean ± SEM. Red dots indicating data points of MET^{N375S}-positive LUSC tumors (*n* = 9). Chi-square (and Fisher's exact) test was performed. (G) Relapse-free survival (RFS) of the 45 LUSC patients with their tumors stained with immunohistochemistry in Figure S8B and C were analyzed with Kaplan-Meier method and log rank test. RFS was measured from time of treatment/surgery to relapse. Subjects who have not reached study-defined endpoint were censored (tick marks) from the analysis (Data cutoff point: January 2018).

Supplementary Figure 11

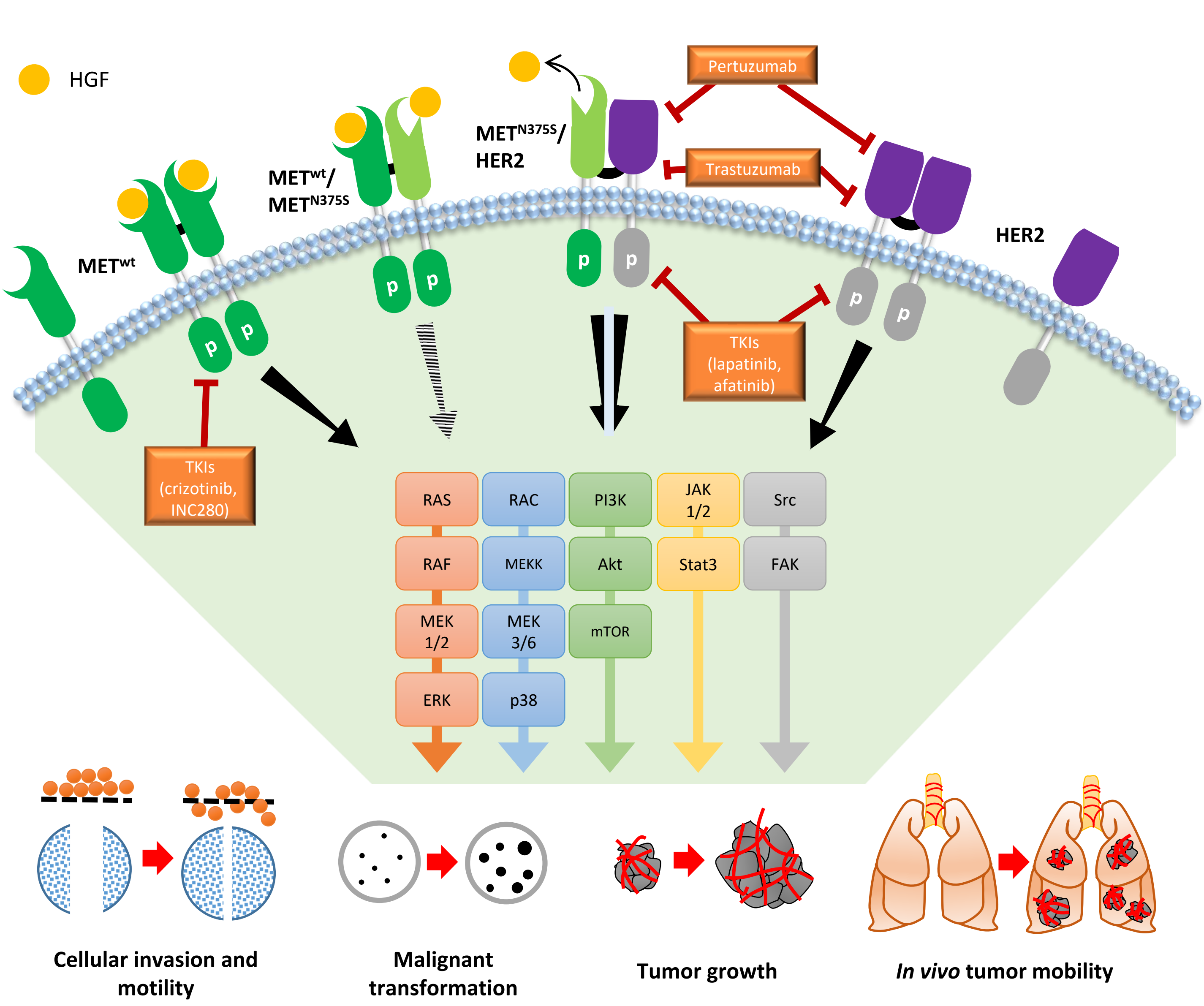

Supplementary Fig. 11: Proposed mechanisms of MET^{N375S} tumors that attribute to aggressive SCC phenotype.

(Left) Wildtype MET requires binding of ligand (HGF) to be activated through receptor dimerization. (Right) Ligand-independent activation of HER2 requires receptor homodimerization, which is a common oncogene addicted pathway in HER2^{+} cells. The *MET* (N375S) polymorphism encodes for a polymorphic receptor which has reduced affinity to HGF, but acquires alternative binding to HER2 through interaction of the MET Sema domain and HER2 D4 domain, leading to tyrosine kinase phosphorylation and downstream activation. These attribute to the aggressive oncogenic phenotype in SCC cells as shown in our study. This oncogenic pathway could be effectively suppressed through inhibition of HER2 either by tyrosine kinase inhibitors (TKIs, eg. afatinib and lapatinib) or monoclonal antibodies (eg. trastuzumab and pertuzumab).

Supplementary Figure 12

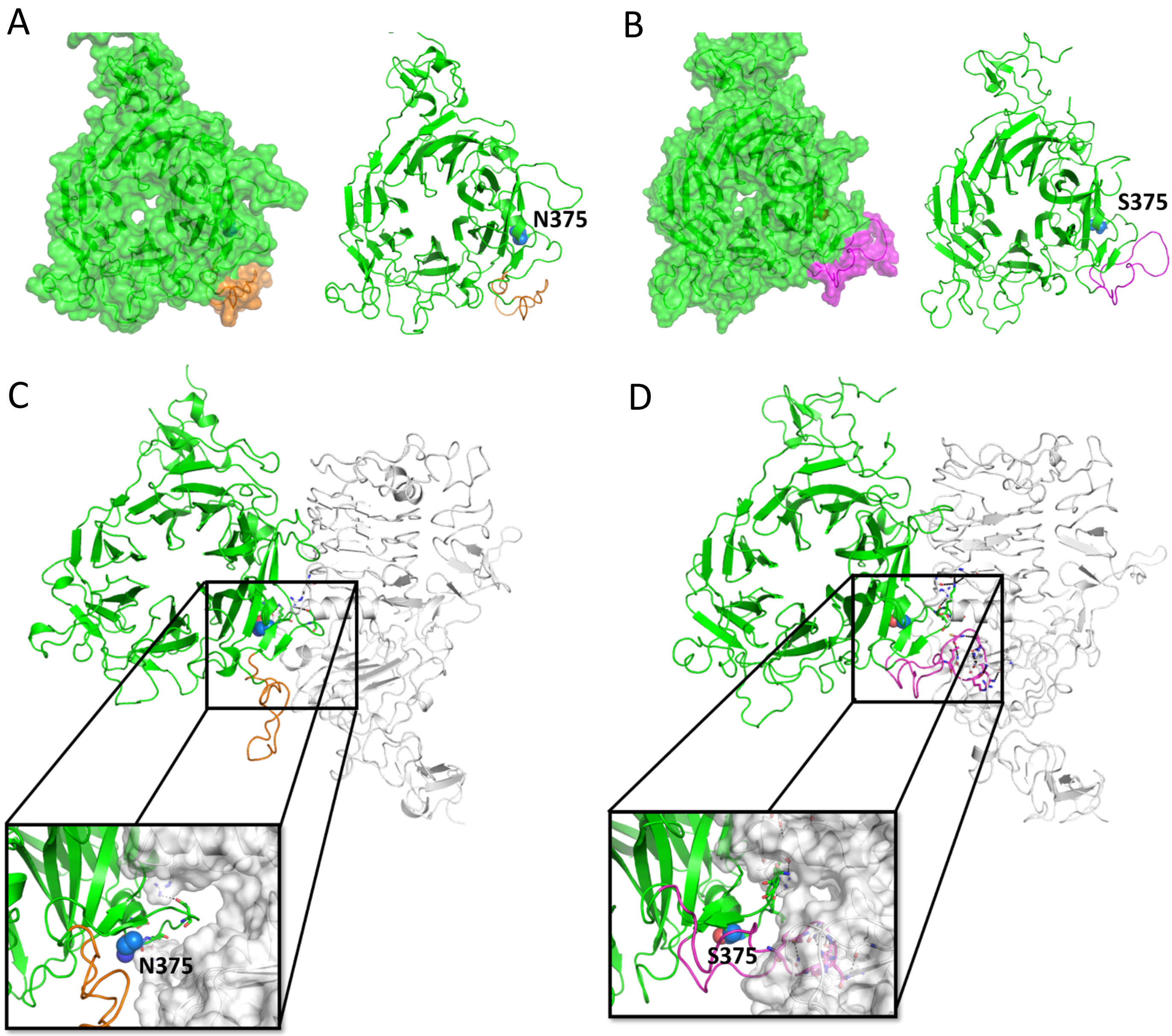

Supplementary Fig. 12: MET-N375S polymorphism is simulated to induce conformation changes to MET, and allowed for better interaction with HER2.
(A-B) A simulated conformation of the SEMA domain of (A) MET^{WT} and (B) MET^{N375S}; the structures are shown in green surface on the left and green ribbon on the right. The regions in the protein that undergo localized conformational changes upon mutation are highlighted in METWT (orange) and MET^{N375S} (magenta) and the site of mutation is shown as spheres (N375 in MET^{wt} and S375 in MET^{N375S}).
(C-D) A model of interaction between the SEMA domain and the extracellular domain of HER2 is shown as cartoon representation in (C) Sema domain of MET^{wt} in green complexed to HER2 in grey and (D) Sema domain of MET^{N375S} in green complexed to HER2 in grey. The region of the Sema domain that undergoes localized conformational changes is highlighted in MET^{wt} (orange) and MET^{N375S} (magenta). A zoomed view of the site of interaction between the Sema domain and HER2 (grey surface) is shown. Black dashed lines indicate interactions between MET and HER2.

Supplementary Table 1

Recombinant Sema + PSI	
rSema^{WT}	IQNVILHEHHIFLGATNYIYVLNEEDLQKVAEYKTGPVLEHPDCFPCQDCSSKANLSGGVWKDNINMALVVDTYYDDQLISCGSVNR<br>GTCQRHVFPHNHTADIQSEVHCIFSPQIEEPSQCPDCVVSALGAKVLSSVKDRFINFFVGNTINSSYFPDHPLHSISVRRLKETKDGFM<br>FLTDQSYIDVLPEFRDSYPIKYVHAFESNNFIYFLTVQRETLDAQTFHTRIIRFCSINSGLHSYMEMPLECILTEKRKKRSTKKEVFNILQA<br>AYVSKPGAQLARQIGASLNDDILFGVFAQSKPDSAEPMDRSAMCAFPIKYVNDFF**N**KIVNKNNVRCLQHFYGPNHEHCFNRTLLR<br>NSSGCEARRDEYRTEFTTALQRVDLFMGQFSEVLLTSISTFIKGDLTIANLGTSEGRFMQVVVSRSGPSTPHVNFLLDSHPVSPEVIVE<br>HTLNQNGYTLVITGKKITKIPLNGLGCRHFQSCSQCLSAPPFVQCGWCHDKCVRSEECLSGTWTQQICL
rSema^{N375S}	IQNVILHEHHIFLGATNYIYVLNEEDLQKVAEYKTGPVLEHPDCFPCQDCSSKANLSGGVWKDNINMALVVDTYYDDQLISCGSVNR<br>GTCQRHVFPHNHTADIQSEVHCIFSPQIEEPSQCPDCVVSALGAKVLSSVKDRFINFFVGNTINSSYFPDHPLHSISVRRLKETKDGFM<br>FLTDQSYIDVLPEFRDSYPIKYVHAFESNNFIYFLTVQRETLDAQTFHTRIIRFCSINSGLHSYMEMPLECILTEKRKKRSTKKEVFNILQA<br>AYVSKPGAQLARQIGASLNDDILFGVFAQSKPDSAEPMDRSAMCAFPIKYVNDFF**S**KIVNKNNVRCLQHFYGPNHEHCFNRTLLRN<br>SSGCEARRDEYRTEFTTALQRVDLFMGQFSEVLLTSISTFIKGDLTIANLGTSEGRFMQVVVSRSGPSTPHVNFLLDSHPVSPEVIVEH<br>TLNQNGYTLVITGKKITKIPLNGLGCRHFQSCSQCLSAPPFVQCGWCHDKCVRSEECLSGTWTQQICL

*Bold text indicates the N375S variation in both recombinant proteins.

Supplementary Table 1: Sequence of recombinant Sema (rSema^{WT} and rSema^{N375S})

Supplementary Table 2

Pathology No	*MET* genotype (WT/N375S)	p-HER2 scoring	p-MET scoring
Case 1	WT	1+	0
Case 2	WT	0	0
Case 3	WT	0	0
Case 4	WT	0	0
Case 5	WT	0	0
Case 6	N375S	2+	1+
Case 7	WT	0	0
Case 8	WT	0	0
Case 9	WT	1+	0
Case 10	WT	2+	1+
Case 11	WT	2+	0
Case 12	WT	0	0
Case 13	WT	0	0
Case 14	WT	3+	0
Case 15	N375S	3+	2+
Case 16	WT	2+	1+
Case 17	N375S	3+	2+
Case 18	WT	0	0
Case 19	N375S	3+	3+
Case 20	N375S	3+	3+
Case 21	WT	1+	1+
Case 22	WT	0	0
Case 23	WT	0	0
Case 24	WT	1+	1+
Case 25	WT	2+	2+
Case 26	WT	1+	1+
Case 27	N375S	3+	2+
Case 28	WT	0	0
Case 29	WT	0	0
Case 30	WT	1+	1+
Case 31	WT	1+	0
Case 32	N375S	3+	2+
Case 33	WT	0	0
Case 34	WT	3+	2+
Case 35	WT	2+	1+
Case 36	WT	0	0
Case 37	N375S	2+	2+
Case 38	WT	2+	1+
Case 39	WT	0	0
Case 40	WT	0	1+
Case 41	N375S	3+	2+
Case 42	WT	1+	2+
Case 43	WT	0	0
Case 44	WT	0	0
Case 45	WT	0	1+

Supplementary Table 2: Scoring of p-HER2 and p-MET in tissue microarray of LUSC.

Supplementary Table 3

guide RNA sequences	
Hs.Cas9.MET.1.AA	/AltR1/rUrU rArCrU rUrCrU rUrGrA rCrGrG rUrCrC rArArA rGrUrU rUrUrA rGrArG rCrUrA rUrGrC rU/AltR2/
Hs.Cas9.MET.1.AF	/AltR1/rArG rGrCrA rUrGrG rArCrA rUrArC rUrUrA rArUrG rGrUrU rUrUrA rGrArG rCrUrA rUrGrC rU/AltR2/)
siRNA sequences	
siMET sequence 1	5'-UGACAUUCUGGAUGGGUGUTG-3'
siMET sequence 2	5'-UUCGAUAUUCAUCACGGCGCG-3'
siERBB2 sequence 1	5'-UGUCUGUGCCGGUGCACACTT-3'
siERBB2 sequence 2	5'-UUCGUCUAAGAUUUCUUUGTT-3'
ddPCR primers and probes	
Forward primer	5'-GCATTCCCTATCAAATATGTC-3'
Reverse primer	5'-GGAGACATCTCACATTGTTT-3'
WT probe	/5HEX/ACTTCTTCA/ZEN/ACAAGATCGTC/3IABkFQ/
N375S probe	/56-FAM/ACTTCTTCA/ZEN/GCAAGATCGT/3IABkFQ/

Supplementary Table 3: Nucleotide sequences for guide RNA, siRNA, ddPCR primers and probes.
