# Supplementary material for: A common MET polymorphism harnesses HER2 signaling to drive aggressive squamous cell carcinoma
Source: Nat Commun. 2020 Mar 25;11:1556. doi: 10.1038/s41467-020-15318-5 (PMC7096530; doi:10.1038/s41467-020-15318-5)
Supplement: Supplementary file 3 — Reporting Summary [file 41467_2020_15318_MOESM3_ESM.pdf]

nature research

Corresponding author(s): Boon Cher Goh and Li Ren Kong

Last updated by author(s): Feb 19, 2020

Reporting Summary

Nature Research wishes to improve the reproducibility of the work that we publish. This form provides structure for consistency and transparency in reporting. For further information on Nature Research policies, see Authors & Referees and the Editorial Policy Checklist .

Statistics

For all statistical analyses, confirm that the following items are present in the figure legend, table legend, main text, or Methods section.

n/a	Confirmed	
☐	☒	The exact sample size (*n*) for each experimental group/condition, given as a discrete number and unit of measurement
☐	☒	A statement on whether measurements were taken from distinct samples or whether the same sample was measured repeatedly
☐	☒	The statistical test(s) used AND whether they are one- or two-sided<br>*Only common tests should be described solely by name; describe more complex techniques in the Methods section.*
☐	☒	A description of all covariates tested
☐	☒	A description of any assumptions or corrections, such as tests of normality and adjustment for multiple comparisons
☐	☒	A full description of the statistical parameters including central tendency (e.g. means) or other basic estimates (e.g. regression coefficient) AND variation (e.g. standard deviation) or associated estimates of uncertainty (e.g. confidence intervals)
☐	☒	For null hypothesis testing, the test statistic (e.g. *F*, *t*, *r*) with confidence intervals, effect sizes, degrees of freedom and *P* value noted<br>*Give P values as exact values whenever suitable.*
☒	☐	For Bayesian analysis, information on the choice of priors and Markov chain Monte Carlo settings
☒	☐	For hierarchical and complex designs, identification of the appropriate level for tests and full reporting of outcomes
☒	☐	Estimates of effect sizes (e.g. Cohen's *d*, Pearson's *r*), indicating how they were calculated

Our web collection on statistics for biologists contains articles on many of the points above.

Software and code

Policy information about availability of computer code

Data collection	NA
Data analysis	Mass spectrometry analyses: The raw files were processed with MaxQuant version 1.5.2.8 (48) with preset standard settings for SILAC labelled samples.<br>Gene expression (RNAseq) analyses: CASAVA v1.8 was used to convert the raw data to fastq files, TopHat2 to map the reads to Ensembl human genome GRCh37, and differentially expressed genes were identified using R package DESeq.<br>Image analyses (PLA, RNA-ISH and IHC): ImageJ Software with Color Deconvolution plugin and Particle Analyzer plugin.<br>structure simulation: Modeller, Pymol, Amber18, CLICK programs

For manuscripts utilizing custom algorithms or software that are central to the research but not yet described in published literature, software must be made available to editors/reviewers. We strongly encourage code deposition in a community repository (e.g. GitHub). See the Nature Research guidelines for submitting code & software for further information.

Data

Policy information about availability of data

All manuscripts must include a data availability statement. This statement should provide the following information, where applicable:

- Accession codes, unique identifiers, or web links for publicly available datasets
- A list of figures that have associated raw data
- A description of any restrictions on data availability

The raw data in this study (mainly RNAseq) has been deposited. GEO Accession number: GSE128956.

Field-specific reporting

Please select the one below that is the best fit for your research. If you are not sure, read the appropriate sections before making your selection.

☒ Life sciences ☐ Behavioural & social sciences ☐ Ecological, evolutionary & environmental sciences

For a reference copy of the document with all sections, see nature.com/documents/nr-reporting-summary-flat.pdf

Life sciences study design

All studies must disclose on these points even when the disclosure is negative.

Sample size	No sample size calculation was applied in this study. Experiments were performed independently for at least 3 times in an unbiased manner.
Data exclusions	No data was purposely omited for the purpose of data analyses.
Replication	All in vitro studies were performed independently by at least two researchers to ensure reproducibility. In vivo validation was conducted by an independent research team.
Randomization	Animals used in this study were randomised into various treatment groups based on their body weight. Each group contained equal distribution of mice with comparable weight.
Blinding	Data analyses were blinded. All images were renamed before batched analyses. Scoring of tumor tissues were conducted independently by a pathologist and a research pathologist independently.

Reporting for specific materials, systems and methods

We require information from authors about some types of materials, experimental systems and methods used in many studies. Here, indicate whether each material, system or method listed is relevant to your study. If you are not sure if a list item applies to your research, read the appropriate section before selecting a response.

Materials & experimental systems

n/a	Involved in the study	
☐	☒	Antibodies
☐	☒	Eukaryotic cell lines
☒	☐	Palaeontology
☐	☒	Animals and other organisms
☒	☐	Human research participants
☒	☐	Clinical data

Methods

n/a	Involved in the study	
☒	☐	ChIP-seq
☒	☐	Flow cytometry
☒	☐	MRI-based neuroimaging

Antibodies

Antibodies used

For immunoblotting, p-MET (Tyr1234/1235, #3077), total Met (#8198), p-HER2 (Tyr1221/1222, #2243), total HER2 (#2165), p-p38 (Thr180/Tyr182, #4511), total p38 (#8690), p-Src (Tyr416, #2101), total Src (#2108), p-ERK1/2 (Thr202/Tyr204, #4377), total ERK1/2 (#4695), p-mTOR (Ser2448, #2971), total mTOR (#2172), p-Stat3 (Tyr705, #9131), total Stat3 (#4904), p-EGFR (Tyr1068, #2234), total EGFR (#2646), p-Akt (Ser473, #4060), total Akt (#9272), lamin B (#13435), α-tubulin (#2125), horseradish peroxidase (HRP)-conjugated Met (#24294), HER2 (#60388), and β-actin (#5125) antibodies were purchased from Cell Signaling Technology; turboGFP antibody was from Origene (#TA150041); pan-Cadherin antibody (#ab22744) from Abcam. Anti-mouse (#7076) and –rabbit (#7074) HRP-conjugated secondary antibodies were obtained from Cell Signaling Technologies. All antibodies were used at 1:2000 dilution.

For co-immunoprecipitation, turboGFP antibody from Origene was used, together with mouse IgG (#5415) antibody from Cell Signaling Technology.

For Duolink PLA assay, rabbit total HER2 was paired with either turboGFP or total MET (Cell Signaling Technology, #8741) of mouse origin, and used at 1:1000 dilution.

For immunofluorescence staining, total HER2 and total MET (Cell Signaling Technology, #8741) were used at 1:1000 dilution; secondary goat anti-mouse Alexa-488 (Thermo Fisher, A-11001 and goat anti-rabbit Alexa-594 (Thermo Fisher, A-11012) were used at 1:1000 dilution.

For immunohistochemistry staining, p-MET and p-HER2 antibodies were used at 1:50 dilution.

Validation

All used antibodies were validaed commercially and reviewed by peers.

Eukaryotic cell lines

Policy information about cell lines

Cell line source(s)	All commercial cell lines were obtained directly from ATCC. Primary HNSCC culture was established and provided by N Gopalakrishna Iyer. HNSCC cell lines (SCC13 and UMSCC1) were provided by H. Phillip Koeffler, and cultured in DMEM medium with standard supplements. Isogenic and CRISPR-cas9 cell lines were generated in-house.
Authentication	All commercial cell lines were authenticated using Promega GenePrint STR method upon delivery to the lab.
Mycoplasma contamination	Mycoplasma contamination test is performed regularly (6-9 months) in our lab. Newly acquired cell lines were tested before experimentation.
Commonly misidentified lines (See ICLAC register)	NA

Animals and other organisms

Policy information about studies involving animals; ARRIVE guidelines recommended for reporting animal research

Laboratory animals	All in vivo xenograft studies were established and maintained in 8- to 10-week-old female SCID mice.
Wild animals	*Provide details on animals observed in or captured in the field; report species, sex and age where possible. Describe how animals were caught and transported and what happened to captive animals after the study (if killed, explain why and describe method; if released, say where and when) OR state that the study did not involve wild animals.*
Field-collected samples	*For laboratory work with field-collected samples, describe all relevant parameters such as housing, maintenance, temperature, photoperiod and end-of-experiment protocol OR state that the study did not involve samples collected from the field.*
Ethics oversight	We adhered to the Institutional Animal Care and Use Committee (IACUC) guidelines on animal use and handling. The patient-derived xenograft is approved by SingHealth Centralised Institutional Review Board (CIRB).

Note that full information on the approval of the study protocol must also be provided in the manuscript.
